# Ternary Complex Components Responsible for Rapid LDL Internalization as Biomarkers for Breast Cancer Associated with Proliferation and Early Recurrence

**DOI:** 10.1158/2767-9764.CRC-23-0562

**Published:** 2025-02-04

**Authors:** Elizabeth S. McDonald, Tien-Chi Pan, Dhruv K. Pant, Melissa A. Troester, Andrew V. Kossenkov, David A. Mankoff, Robert H. Mach, Lewis A. Chodosh

**Affiliations:** 1 Division of Breast Imaging, Department of Radiology, Perelman School of Medicine at the University of Pennsylvania, Philadelphia, Pennsylvania.; 2 Department of Cancer Biology, Perelman School of Medicine at the University of Pennsylvania, Philadelphia, Pennsylvania.; 3 Abramson Family Cancer Research Institute, Perelman School of Medicine at the University of Pennsylvania, Philadelphia, Pennsylvania.; 4 Department of Epidemiology, UNC Gillings School of Global Public Health, Chapel Hill, North Carolina.; 5 Center for Systems and Computational Biology, The Wistar Institute, Philadelphia, Pennsylvania.; 6 Division of Nuclear Medicine and Molecular Imaging, Department of Radiology, Perelman School of Medicine at the University of Pennsylvania, Philadelphia, Pennsylvania.; 7 Radiochemistry, Department of Radiology, Perelman School of Medicine at the University of Pennsylvania, Philadelphia, Pennsylvania.

## Abstract

The ternary complex of progesterone receptor membrane component 1 (PGRMC1)–sigma-2 receptor/transmembrane protein 97 (σ2R/TMEM97)–low-density lipoprotein receptor (LDLR) has recently been discovered and plays a role in cholesterol transport. This study investigated whether individual components of that complex are prognostic breast cancer biomarkers and have defined expression in established molecular subtypes. A total of 4,463 invasive breast cancers were analyzed as a function of molecular and phenotypic markers, estimates of cellular proliferation, and recurrence-free survival. A gene expression signature–based assay was utilized to estimate cellular proliferation. Cox proportional hazards regression estimated relapse-free survival and multivariate Cox analysis adjusted for the association of proliferation with early relapse. *PGRMC1–σ2R/TMEM97–LDLR* expression was stratified by immunohistochemical (IHC) and molecular subtype, tumor grade, and size. *TMEM97* exhibited the strongest correlation with proliferation, highest in estrogen receptor (ER)–positive disease (*r* = 0.59, *P* = 8.1^−114^). *TMEM97* and *PGRMC1* were associated with a risk of early recurrence, dependent upon their association with proliferation. The risk of early recurrence was highest with *TMEM97* and only seen in ER+/HER2− disease [HR = 1.5; 95% confidence interval (CI) = 1.35–1.67; *P* = 5.4^−14^] and ER+ malignancies (HR = 1.49; 95% CI = 1.31–1.68; *P* = 3.1^−10^). There was no increased risk of recurrence with TMEM97 expression in ER−/HER2− (HR = 1.05; 95% CI = 0.88–1.25; *P* = 0.63) or ER− disease (HR = 1.02; 95% CI = 0.89–1.17; *P* = 0.75). Components of a ternary complex associated with rapid internalization of low-density lipoprotein are biomarkers associated with cellular proliferation and early recurrence, which should help guide studies exploring them in the context of additional markers of aggressive disease. Elucidating the role of PGRMC1, TMEM97, and LDLR in breast cancer will facilitate a mechanistic understanding of how proliferation interplays with cholesterol metabolism in malignant transformation or propagation.

**Significance:**

This first large-scale analysis of the putative ternary complex responsible for rapid low-density lipoprotein internalization in breast cancer reveals a link between component expression and recurrence, with prognostic implications for identifying patients needing supplemental posttreatment surveillance and/or additional therapeutic approaches.

## Introduction

Cholesterol is an essential component of cell membranes, and its metabolism is often altered in cancer (1). Although some studies have not found a significant link between lipoproteins and breast cancer, others have identified a correlation between low-density lipoprotein (LDL) cholesterol levels and breast cancer risk (2). A recent large analysis indicates that elevated levels of both high-density lipoprotein (HDL) and LDL cholesterol are associated with an increased risk of breast cancer (3), and additional investigations are ongoing.

Statins, which are used to lower cholesterol levels, have been investigated for their potential role in cancer treatment and recurrence. However, the results have been inconsistent, and no definitive benefits have been established (4–7). The biochemical pathways of cholesterol are complicated, including biosynthesis and uptake through the LDL receptor (LDLR) pathway. Statins downregulate cholesterol production by the liver (preventing biosynthesis). Still, if cancer cells have other ways to get cholesterol, then the cell may be able to circumvent the lower production levels.

Selective estrogen receptor modulators (SERM), such as tamoxifen, are estrogen receptor (ER) antagonists that have long been used for the treatment of patients with ER+ breast cancer. However, tamoxifen and other SERM can inhibit angiogenesis, independent of their inhibitory effect on ERs (8). One molecular mechanism that allows SERM to inhibit angiogenesis is inhibiting cholesterol trafficking in endothelial cells (9). In endothelial cells, VEGFR2 and mTOR are major signaling proteins that are regulated by cholesterol levels (10, 11). The inhibitory effects of SERM on VEGFR2 and mTOR signaling, as well as angiogenesis, were rescued by replenishing endothelial cells with cholesterol, suggesting that inhibition of cholesterol trafficking is a primary effect of SERM for + antiangiogenic activity (12). Although the cholesterol trafficking inhibition is ER independent, the exact molecular target is still unknown.

There are four proteins related to progesterone receptor membrane component (PGRMC) that have a cytochrome b5–like heme/sterol-binding domain, but of these, only PGRMC1 is known to bind progesterone (P4) in the low nanomolar range (13). PGRMC1 may be related to both breast cancer proliferation and cholesterol transport. PGRMC1 facilitates triple-negative breast cancer tumor growth *in vivo* (14), and in an ER+ human breast cancer cell line that overexpresses PGRMC1, medroxyprogesterone acetate and norethisterone treatment significantly increased proliferation (15, 16). PGRMC1 may also be associated with breast cancer chemotherapeutic resistance *in vitro*. Doxorubicin-mediated apoptosis was decreased by 50% when a PGRMC1 triple-negative breast cancer cell line was pretreated with progesterone. PGRMC1-depleted cells lost the progesterone-mediated survival advantage (14). Thus, PGRMC1 could be an important breast cancer biomarker.

Sigma-2 receptor/transmembrane protein 97 (σ2R/TMEM97) is a protein involved in cholesterol homeostasis and regulation of cell growth found in cellular membranes (17), lipid rafts (18), endoplasmic reticulum, lysosomes, and plasma membranes (19). σ2R density is high in multiple cancers (20–22), and σ2R levels are elevated in aldehyde dehydrogenase (ALDH)–high compared with ALDH-low MDA-MB-435 cells. The ALDH phenotype has been reported as a surrogate marker for tumor-initiating cells (cancer stem cells; ref. 23). Elevated σ2R levels are found in lung tumors and plasma from patients with lung cancer (24), and preclinical evidence suggests that σ2R may be a therapeutic target as σ2R ligands potentiate the efficacy of chemotherapeutic agents in mouse models of pancreatic cancer and improve survival (25–27). Studies have shown that σ2R/TMEM97 ligands may also be useful in the treatment of a number of neurologic disorders, including Huntington disease (28), neuropathic pain (29), and Alzheimer disease (30). As a result, a diverse set of σ2R/TMEM97 radiotracers and ligands has been developed for use in strategies targeting cancer diagnosis and treatment (31). One such radiotracer is the σ2R-selective radioligand imaging agent N-(4-(6,7-dimethoxy-3,4-dihydroisoquinolin-2(1H)-yl)butyl)-2-(2-^18^F-fluoroethoxy)-5-methylbenzamide (^18^F-ISO-1; ref. 32). The ability of this imaging agent to measure both σ2R density and cellular proliferation has been validated in preclinical models (33) and in early results in a variety of solid tumors (34). *In vitro* work has demonstrated that sigma-2 agents can be used as alternative tracers for proliferation in breast cancer cell lines (35).

Our lab has previously linked these two important proteins by showing that PGRMC1 and σ2R/TMEM97 form a complex with the LDLR, and the intact complex is required for efficient uptake of lipoproteins such as LDL (36). This complex represents a common biological mechanism for cholesterol uptake in a variety of cells including neurons (37) and breast cancer cells (manuscript in preparation). Supporting this, siRNA studies knocking down TMEM97 demonstrated a reduction in the rate of internalization of LDL by the LDLR (38). We also demonstrated in a subsequent clinical trial that *in vivo* quantification of a radioligand targeting TMEM97 correlates with proliferation in ER+ breast cancer (39). Although PGRMC1 is a membrane-associated progesterone receptor, its role in cell biology is historically poorly understood. It is likely a molecular chaperone that is involved in the translocation of lipophilic molecules such as cholesterol and other steroids from the plasma membrane and the endoplasmic reticulum, mitochondria, and other organelles. Before its identification as TMEM97, the σ2R had also been implicated in cholesterol biosynthesis. Although we have demonstrated that PGRMC1 and σ2R/TMEM97 are involved in the same biochemical pathways within the cell, little is known about the impact of the individual components on breast cancer clinical outcomes. The high association of PGRMC1 and σ2R/TMEM97 and the suspected role of both proteins in proliferation spurred this investigation.

A possible link between cholesterol metabolism and ER+ breast cancer has been considered for decades, but the mechanism has been elusive, limiting possible therapeutic interventions for risk modification. Building on the studies above, our clinical question was whether components of the PGRMC1–TMEM97–LDLR protein complex affect clinical outcomes in breast cancer. To accomplish this on a large scale, we linked 17 publicly available databases. We also validated a new proliferation signature to allow adjustment for the clinically suspected link between this complex and proliferation in breast cancer because standard measures of proliferation like Ki-67 expression were not available in these datasets. Although the PGRMC1–TMEM97–LDLR protein complex could be a potential diagnostic or therapeutic target, little is known about the *in vivo* expression of these proteins in subtypes of human breast cancer or their association with clinical outcomes. We tested the hypothesis that these proteins correlate with proliferation in human breast cancer in order to examine the relationship between proteins affecting cholesterol transport and breast cancer subtypes, cellular proliferation, and markers of proliferation. We also evaluated the association among *PGRMC1*, *TMEM97*, *LDLR*, and breast cancer recurrence to determine whether they are prognostic biomarkers for aggressive disease.

## Materials and Methods

### Human breast cancer microarray datasets

A multiple-platform data integration method was utilized to normalize and simultaneously analyze microarray data from 17 publicly available primary breast cancer microarray datasets (“Integrated Dataset,” Supplementary Table S1). Microarray data and corresponding clinical annotations were downloaded from NCBI Gene Expression Omnibus (RRID: SCR_005012) or the original authors’ websites. Microarray data were converted to a log_2_ scale where necessary. Affymetrix microarray data were renormalized using robust multiarray average when .CEL files were available. Five breast cancer subtypes were used according to the PAM50 classification (40). PAM50 is a gene expression assay that can be used to categorize breast tumors into intrinsic subtypes that indicate distinct tumor behaviors. In total, data were available from 4,463 invasive breast cancers: 1,164 luminal A, 921 luminal B, 645 HER2-enriched, 860 basal, and 543 normal-like. In four datasets, patients received no systemic treatment; in two datasets, patients received neoadjuvant treatment; and the remaining datasets represented a mixture of adjuvant and no treatment (Supplementary Table S2).

### Gene expression and prognostic variables/subtypes

The association between mRNA expression and categorical prognostic variables in human breast cancers, including ER status, progesterone receptor (PR) status, HER2 status, lymph node involvement, tumor size, tumor grade, and intrinsic molecular subtype, was assessed by ANOVA in pooled microarray datasets. For each categorical prognostic variable, gene expression was normalized against the mean expression of the same baseline group in each dataset and pooled across all datasets for which the prognostic variable was available. Baseline normalization was performed by subtracting mean gene expression (log_2_ scale) in the baseline group from gene expression in each sample.

### The Cancer Genome Atlas data analysis

The Cancer Genome Atlas (TCGA; RRID: SCR_003193) was queried for invasive breast cancers with available RNA sequencing data (RNA Seq V2 RSEM) and differentiated according to IHC subtype. In total, data were available from 1,019 invasive breast cancers (Supplementary Table S3): 738 ER+, 215 ER−, 643 PR^+^, 307 PR^−^, 149 HER2+, 508 HER2−, and 102 triple-negative breast cancer (TNBC; ER−/PR^−^/HER2−). *PGRMC1* expression was first compared between all groups and plotted according to subtype. *PGRMC1* was then tested for correlation with 20,531 genes, which resulted in the identification of 461 genes in which expression was correlated with *PGRMC1* expression (Pearson *r* > 0.25) within tumor samples. A heatmap of expression levels for the top positively and negatively correlated genes was generated. These were analyzed for enrichment of pathways, functions, networks, and upstream regulators using Ingenuity Pathway Analysis (RRID: SCR_008653; QIAGEN Redwood City, www.qiagen.com/ingenuity; Supplementary Table S4). The analysis was done using functions from MATLAB R2012b (RRID: SCR_001622).

### Proliferation gene expression signature

Measures of proliferation, such as Ki-67 expression or mitotic index, were not available for the majority of breast cancer samples. Therefore, to estimate relative proliferation levels in human breast cancer samples, we generated a gene expression signature containing 224 genes (Prolif224) from the overlap of two gene sets: (i) 651 cell cycle–regulated genes identified in HeLa cells (41) and (ii) 1,882 serum-responsive genes identified in human fibroblasts (42). Serum-responsive genes were identified by differential expression analysis between the 0.1% and 10% serum groups using Cyber-T (43) at an FDR of 10%. In each human breast cancer dataset, levels of proliferation were estimated using these 224 genes and a previously described scoring method (44), in which each gene was weighted using its log fold change between the 0.1% and 10% serum groups (42).

Correlation between the expression of individual genes and estimated relative proliferation level was assessed in human breast cancer datasets using the Pearson correlation coefficient and summarized across datasets by meta-analysis. Additional meta-analyses of Pearson correlation were performed in subsets of samples stratified by ER status, HER2 status, lymph node status, or molecular subtype. Correlation between two different genes was assessed in a similar fashion.

### Gene expression and relapse-free survival

Within each dataset, the effect size of the association between mRNA expression and 5-year relapse-free survival was estimated using the HR from Cox proportional hazards regression in which gene expression was modeled as a continuous variable. Effect size estimates were combined across datasets by meta-analysis using the inverse variance weighting method (45). Between-study homogeneity of survival association was tested using the χ^2^ test on Cochran’s Q statistic (46), for which a *P* value of less than 0.05 was interpreted as evidence of significant heterogeneity. In the presence of significant heterogeneity, the random-effects model (47) was used for meta-analysis. In the absence of significant heterogeneity, the fixed-effects model (48) was used. Cox proportional hazards regression and meta-analysis were performed using the “coxph” function in the “survival” package and the “metagen” function in the “meta” packages in R 2.15.0. For datasets in which relapse-free survival information was not available, distant metastasis-free survival or disease-specific survival information, when available, was used for survival analysis.

Additional meta-analyses were performed in subsets of samples stratified by ER status, HER2 status, lymph node status, or intrinsic molecular subtype, as well as in the subset of patients who, according to available treatment information, did not receive any adjuvant systemic treatment. As HER2 IHC status was not available for several datasets, HER2 status was approximated by *ERBB2* mRNA expression as measured by microarray in a similar fashion as the Cancer Outlier Profile Analysis (49). In each dataset, HER2+ and HER2− samples were defined as being above and below a cutoff of 1.5 absolute deviations above the median, respectively, which resulted in an average specificity of 98% and sensitivity of 78% in five validation datasets (50–54). Due to the nonrandom association between ER and HER2 status, an approximation of HER2 status was not attempted in datasets consisting entirely of hormone receptor–positive or hormone receptor–negative cancers. Assignments of intrinsic subtype were done using the PAM50 classifier (40) after expression data were median-centered for each gene.

### Data availability

The data generated in this study are available upon request from the corresponding authors.

## Results

### PGRMC1, TMEM97, and LDLR are overexpressed in ER− breast cancer

**Figure 1:**
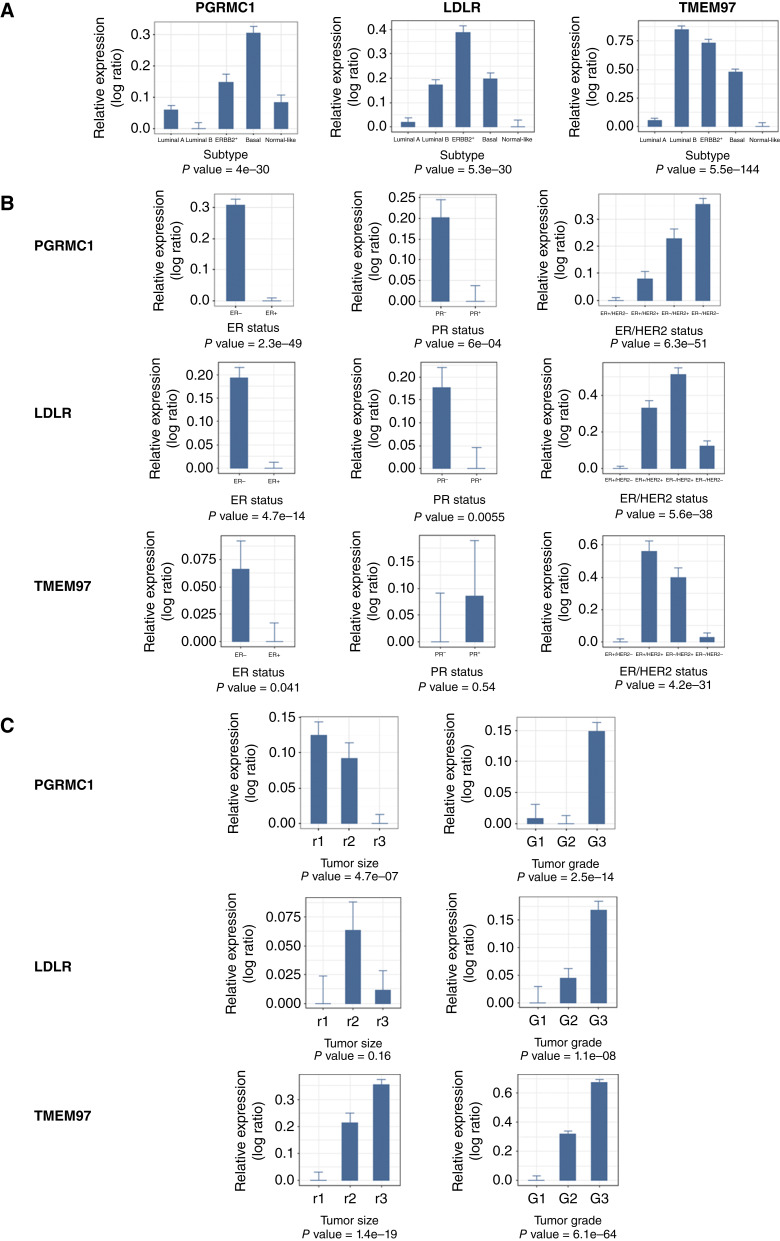
**A,** PGRMC1 is overexpressed in human breast cancers of the basal subtype using PAM50. TMEM97 has the highest expression in luminal B tumors. **B,** PGRMC1 is overexpressed in hormone receptor–negative cancers. LDLR expression is highest in ER−/HER2+. TMEM97 has the highest expression in ER+/HER2+ tumors. **C,** PGRMC1 is overexpressed in smaller higher-grade tumors. LDLR and TMEM97 expression is highest in higher-grade tumors.

Publicly available microarray data for 4,463 patients contained within 17 human primary breast cancer datasets (50–52, 55–67), along with the corresponding clinical annotations, were downloaded and converted to a log_2_ scale where necessary. Affymetrix microarray data for which .CEL files were available were renormalized using robust multiarray average (68). PGRMC1 is overexpressed in human breast cancers of the basal subtype using PAM50 (*P* = 4 × 10^−30^). TMEM97 has the highest expression in luminal B tumors (Fig. 1A). In an analogous manner, *PGRMC1* was expressed at higher levels in ER− tumors, PR^−^ tumors, and ER−/HER2− tumors (Fig. 1B). *PGRMC1* was also expressed at higher levels in tumors of higher grade (*P* = 2.5 × 10^−14^) and smaller size (*P* = 4.7 × 10^−7^; Fig. 1C). In the integrated dataset, *LDLR* and *TMEM97* also had the highest expression in ER− disease (*P* = 4.7e−14 and *P* = 0.041, respectively; Fig. 1B); *LDLR* and *TMEM97* were also each expressed at higher levels in tumors of higher grade (*P* = 1.1 × 10^−08^ and 6.1 × 10^−64^, respectively; Fig. 1C).

To examine *PGRMC1* expression in cancers compared with normal breast tissue, TCGA data were analyzed based on tumor IHC classification for 738 ER+, 215 ER−, 643 PR^+^, 307 PR^−^, 149 HER2+, 508 HER2−, and 102 TNBC tumors and 108 normal controls. Normal tissues in TCGA database are matched samples (normal tissue from patients who also have a primary tumor). Consistent with its elevated expression in the basal subtype, *PGRMC1* was overexpressed in ER−, PR^−^, and TNBC compared with normal breast tissue (1.33-fold, *P* = 2 × 10^−06^; 1.23-fold, *P* = 3 × 10^−04^; and 1.30-fold, *P* = 6 × 10^−6^, respectively; Supplementary Fig. S1).

### PGRMC1 and TMEM97 expression are associated with cellular proliferation in breast cancer

The association between components of the ternary complex and cellular proliferation in human breast cancer was assessed, in comparison with *TK1* as a validated marker for cellular proliferation (69). Because the vast majority of clinical samples for which *PGRMC1* expression was available did not have documented measures of cellular proliferation, such as Ki-67 or mitotic index, we pursued a computational approach utilizing a gene expression signature for proliferation to estimate cellular proliferation rates. First, we generated a gene expression signature containing 224 genes from the overlap of gene sets representing 651 cell cycle–regulated genes in HeLa cells (41) and 1,882 serum-responsive genes in human fibroblasts (see “Materials and Methods”; ref. 42). Next, in each human breast cancer dataset, levels of proliferation were estimated for each sample using this 224-gene set in combination with a previously described scoring method (44) in which each gene was weighted using its log fold change between the 0.1% and 10% serum groups (42).

*PGRMC1* exhibited a robust positive association with signature-derived proliferation scores across all breast cancers (*r* = 0.268; *P* = 6.5 × 10^−17^; Table 1). When PAM50 molecular subtypes were considered, *PGRMC1* displayed a significant correlation with proliferation scores within each of the five subtypes, with the strongest association observed for the basal subtype (*r* = 0.415; *P* = 2.4 × 10^−37^). *PGRMC1* expression was also correlated with proliferation within each receptor subtype, except for ER+/HER2+ tumors (Table 1). As such, PGRMC1 was a consistent marker for proliferation across subtypes, significantly correlating with proliferation in HER2+, ER−/HER2+, ERBB2-enriched, and luminal B tumors. *PGRMC1* expression was significantly associated with proliferation scores within lymph node–positive and lymph node–negative tumors (Table 1). In the PGRMC1–σ2R/TMEM97–LDLR complex, *TMEM97* exhibited the strongest correlation with proliferation and the highest in ER+ disease (all: *r* = 0.509; *P* = 6.1e^−67^ and ER+: *r* = 0.588; *P* = 8.1^−114^), and *LDLR* only had a weak correlation with proliferation, regardless of subtype or IHC status (all: *r* = 0.16; *P* = 6.6^−11^; Table 1).

**Table 1:** Association of PGRMC1, TK1, LDLR, and TMEM97 with proliferation. Meta-analysis was performed to examine the association among a) *PGRMC1*, b) *TMEM97*, c) *TK1*, and d) *LDLR* and estimated proliferation rates of tumors

Strata	Correlation coefficient	*P* value	Strata	Correlation coefficient	*P* value
a) PGRMC1 vs. proliferation signature			b) TMEM97 vs. proliferation signature		
All	**0.268**	**6.50e−17**	All	**0.509**	**6.1e−67**
ER+	**0.15**	**0.00017**	ER+	**0.588**	**8.1e−114**
ER−	**0.289**	**1.00e−10**	ER−	**0.38**	**8.2e−38**
HER2+	**0.204**	**0.0021**	HER2+	**0.392**	**1.5e−22**
HER2−	**0.271**	**1.40e−14**	HER2−	**0.524**	**1.6e−101**
ER+/HER2+	0.098	0.11	ER+/HER2+	**0.422**	**5.6e−13**
ER+/HER2−	**0.115**	**0.0019**	ER+/HER2−	**0.587**	**1.1e−110**
ER−/HER2+	**0.279**	**0.0086**	ER−/HER2+	**0.399**	**1.4e−10**
ER−/HER2−	**0.279**	**5.50e−15**	ER−/HER2−	**0.493**	**3.1e−47**
Node^+^	**0.302**	**1.10e−09**	Node^+^	**0.486**	**6.7e−30**
Node^−^	**0.221**	**7.20e−13**	Node^−^	**0.472**	**1.5e−38**
Basal	**0.415**	**2.40e−37**	Basal	**0.43**	**1.2e−16**
ERBB2-enriched	**0.254**	**1.70e−05**	ERBB2-enriched	**0.385**	**1.7e−10**
Luminal A	**0.07**	**0.018**	Luminal A	**0.394**	**2.7e−44**
Luminal B	**0.102**	**0.0021**	Luminal B	**0.486**	**2.8e−56**
Normal-like	**0.091**	**0.038**	Normal-like	**0.501**	**1.9e−35**
c) TK1 vs. proliferation signature			d) LDLR vs. proliferation signature		
All	**0.688**	**2.50e−145**	All	**0.16**	**6.6e−11**
ER+	**0.712**	**6.50e−200**	ER+	**0.176**	**2.4e−10**
ER−	**0.531**	**4.00e−27**	ER−	**0.043**	**0.16**
HER2+	**0.445**	**4.80e−31**	HER2+	**0.137**	**0.00082**
HER2−	**0.725**	**6.60e−177**	HER2−	**0.154**	**2.3e−07**
ER+/HER2+	**0.528**	**2.10e−21**	ER+/HER2+	**0.159**	**0.0098**
ER+/HER2−	**0.727**	**1.40e−202**	ER+/HER2−	**0.155**	**6.7e−06**
ER−/HER2+	**0.338**	**4.20e−08**	ER−/HER2+	**0.143**	**0.025**
ER−/HER2−	**0.616**	**2.00e−27**	ER−/HER2−	**0.086**	**0.019**
Node^+^	**0.688**	**1.30e−44**	Node^+^	**0.137**	**2.9e−08**
Node^−^	**0.692**	**1.00e−150**	Node^−^	**0.176**	**5.8e−19**
Basal	**0.574**	**4.70e−30**	Basal	**0.1**	**0.0039**
ERBB2-enriched	**0.365**	**1.80e−21**	ERBB2-enriched	**0.136**	**0.00069**
Luminal A	**0.551**	**5.00e−32**	Luminal A	**0.143**	**0.0017**
Luminal B	**0.524**	**3.00e−68**	Luminal B	**0.115**	**0.00051**
Normal-like	**0.636**	**2.00e−65**	Normal-like	**0.137**	**0.0017**

*P* value < 0.05.

The association of proliferation scores with *TK1* expression was also analyzed, given its known positive correlation with proliferation. *TK1* is a cell cycle–regulated target of E2F in which expression and function are associated with cell-cycle status. *TK1* expression also correlates with the uptake of 3′-deoxy-3′-[^18^F]fluorothymidine (^18^F-FLT; refs. 70, 71). ^18^F-FLT is trapped in cells after undergoing phosphorylation by *TK1*, which is catalytically active during S-phase and represents the first metabolic step in the salvage pathway for incorporating exogenous thymidine into DNA (72–74). ^18^F-FLT is currently the most widely used radiotracer for imaging tumor proliferation rates (75–77) with uptake reflecting *ex vivo* S-phase–specific bromodeoxyuridine incorporation and TK expression. ^18^F-FLT was demonstrated to be a useful biomarker for breast cancer treatment response in a large multicenter trial (78). In our study, *TK1* exhibited a strong positive association with proliferation scores (*r* = 0.688; *P* = 2.5 × 10^−145^), particularly within ER+/HER2− tumors (*r* = 0.727; *P* = 1.4 × 10^−202^; Table 1).

### PGRMC1 and TMEM97 expression are associated with early breast cancer relapse

**Figure 2:**
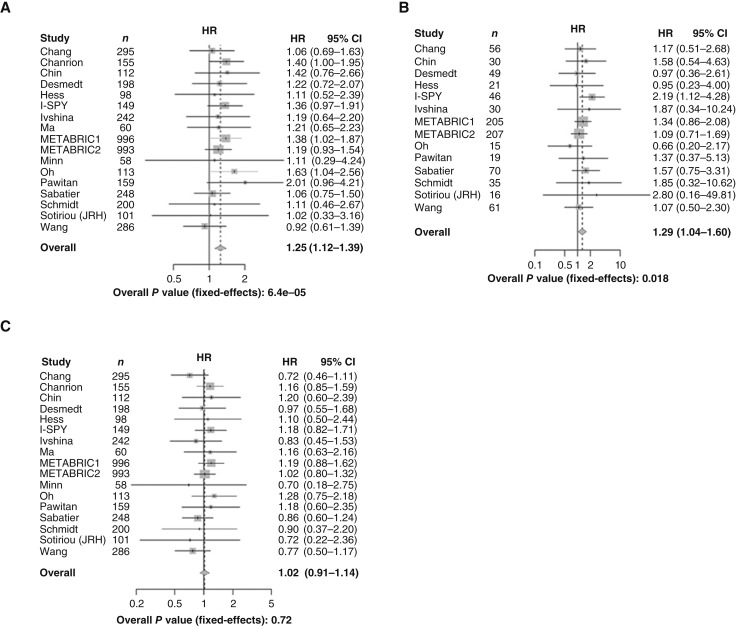
PGRMC1 is associated with early breast cancer relapse in a proliferation-dependent manner. Effect size estimates were aggregated across datasets by meta-analysis to determine the risk of relapse within 5 years from all cancers. **A,** Association of PGRMC1 with early breast cancer relapse. **B,** Association of PGRMC1 with early breast cancer relapse within the basal subtype. **C,** Association of PGRMC1 with early relapse adjusted for estimated proliferation. JRH, John Radcliffe Hospital.

**Figure 3:**
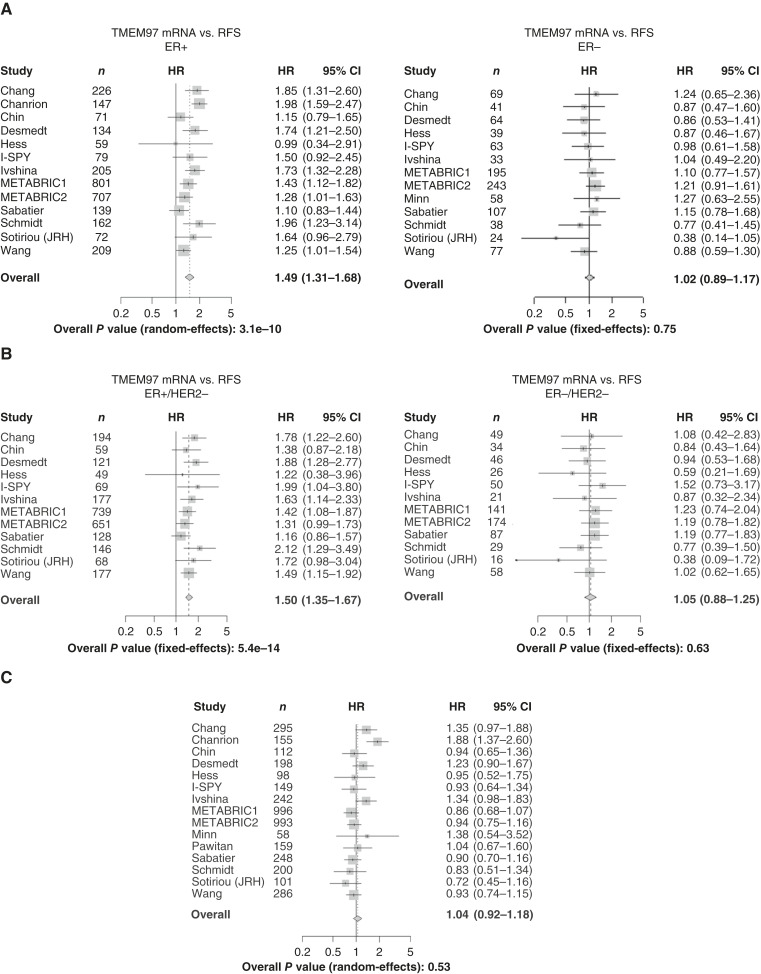
*TMEM97* is associated with early breast cancer relapse in a proliferation-dependent manner. Effect size estimates were aggregated across datasets by meta-analysis to determine the risk of relapse within 5 years. **A,** TMEM97 is associated with early breast cancer relapse only in ER+ tumors. **B,** TMEM97 is associated with early breast cancer relapse only in ER+/HER2− tumors. **C,** Association of TMEM97 with early breast cancer relapse in ER+/HER2− tumors adjusted for proliferation. JRH, John Radcliffe Hospital; RFS, recurrence-free survival.

To address whether components of the ternary complex were associated with the risk of breast cancer relapse, effect size estimates from Cox proportional hazards regression using gene expression as a continuous variable were aggregated across datasets by meta-analysis. The results demonstrated that *PGRMC1* expression is associated with a higher risk of early relapse (within 5 years) across all patients with breast cancer [HR = 1.25; 95% confidence interval (CI) = 1.12–1.39; *P* = 6.4 × 10^−5^; Fig. 2A]. Within the basal subtype, *PGRMC1* expression was also associated with relapse (HR = 1.29; 95% CI = 1.04–1.60; *P* = 0.018; Fig. 2B). The risk of early recurrence with *TMEM97* was present only in ER+/HER2− disease (HR = 1.5; 95% CI = 1.35–1.67; *P* = 5.4^−14^) and ER+ malignancies (HR = 1.49; 95% CI = 1.31–1.68; *P* = 3.1^−10^) and was not present in ER−/HER2− (HR = 1.05; 95% CI = 0.88–1.25; *P* = 0.63) or ER− disease (HR = 1.02; 95% CI = 0.89–1.17; *P* = 0.75; Fig. 3A and B). *LDLR* was not associated with a risk of early recurrence in ER+ disease (HR = 0.99; 95% CI = 0.87–1.13; *P* = 0.93) or ER+/HER2− tumors (HR = 1.01; 95% CI = 0.87; 1.17; *P* = 0.9).

The association of *TK1* expression with recurrence-free survival was tested to evaluate whether the association of *PGRMC1* and *TMEM97* expression with recurrence-free survival might be linked to their association with proliferation. Expression of *TK1* was associated with decreased relapse-free survival overall (HR = 1.45; 95% CI = 1.32–1.60; *P* = 3.4 × 10^−14^), particularly within the luminal A subtype (HR = 1.81; 95% CI = 1.29–2.54; *P* = 5.4 × 10^−4^) but not in the basal subtype (HR = 1.14; 95% CI = 0.97–1.34; *P* = 0.13; Supplementary Fig. S2). In contrast, *PGRMC1* was associated with decreased relapse-free survival overall (Fig. 2A), with no effect in the luminal A subtype (HR = 1.03; 95% CI = 0.72–1.49; *P* = 0.86). *TK1* expression was also associated with an increased risk of recurrence in combined ER+/HER2− tumors (HR = 1.67; 95% CI = 1.49–1.88; *P* = 5.1 × 10^−18^) as well as in ER+ and HER2− tumors (Supplementary Fig. S3).

After adjusting for estimated tumor proliferation rates, *PGRMC1*, *TMEM97*, and *TK1* were not associated with relapse-free survival (HR = 1.02; 95% CI = 0.91–1.14; HR = 1.04; 95% CI = 0.92–1.18; and HR = 1.05; 95% CI = 0.95–1.15, respectively; Figs. 2C and 3C; Supplementary Fig. S4]. This suggests that the associations of *PGRMC1*, *TMEM97*, and *TK1* with relapse-free survival are each mediated by their respective associations with cellular proliferation.

### PGRMC1 expression is weakly associated with TK1 expression

As the expression of *PGRMC1* and *TK1* are associated with proliferation in human breast cancers, we next asked whether *PGRMC1* expression was associated with the expression of *TK1*. *PGRMC1* exhibited a significant correlation with *TK1* when all cancers were combined, although the magnitude of these associations was weak (*r* = 0.154; *P* = 9 × 10^−08^; Table 2). Somewhat stronger associations were observed between *PGRMC1* and *TK1* expression within the basal subtype (*r* = 0.241; *P* = 1.2 × 10^−12^) and within ER−/HER2− breast cancers (*r* = 0.256; *P* = 9.2 × 10^−13^); however, the magnitude of these associations was smaller than the correlation between *PGRMC1* and proliferation scores within these same subsets of patients. This suggests that the association between *PGRMC1* and cellular proliferation is largely independent of the association between *PGRMC1* expression and expression of *TK1*.

**Table 2:** *PGRMC1* is weakly associated with *TK1*. PGRMC1 exhibits a significant correlation with TK1 when all cancers are combined, although the magnitude of these associations is weak. *PGRMC1* vs. *TK*1

Strata	Correlation coefficient	*P* value
All	**0.154**	**9.00E**−**08**
ER+	0.016	0.66
ER−	**0.214**	**7.90E**−**13**
HER2+	**0.102**	**0.013**
HER2−	**0.141**	**8.50E**−**07**
ER+/HER2+	0.009	0.88
ER+/HER2−	−0.037	0.059
ER−/HER2+	**0.147**	**0.021**
ER−/HER2−	**0.256**	**9.20E**−**13**
Node^+^	**0.158**	**0.00017**
Node^−^	**0.105**	**1.50E**−**07**
Basal	**0.241**	**1.20E**−**12**
ERBB2^+^	**0.212**	**8.50E**−**08**
Luminal A	−0.063	0.15
Luminal B	0.009	0.79
Normal-like	−0.015	0.73

*P* value < 0.05.

### PGRMC1 expression is associated with the activation of cell-cycle pathways

TCGA data containing 1,019 breast cancers were analyzed in an exploratory fashion to evaluate expression patterns associated with *PGRMC1*. A total of 20,531 available genes were tested for correlation with *PGRMC1*. Within this dataset, 461 genes were significantly associated with *PGRMC1* with a coefficient of at least 0.25 (Supplementary Fig. S5A). These genes were analyzed for enrichment of pathways and targets for upstream regulators using Ingenuity Pathway Analysis. *PGRMC1* was associated with *CCND1* (cyclin D1) and *MYC* target pathway activities (*z* = 2.45; *P* = 8 × 10^−5^ and *z* = 1.95; *P* = 10^−6^, respectively) and *RICTOR* target pathway inhibition (*z* = −4; *P* = 3 × 10^−5^; Supplementary Table S4). Exploratory pathway enrichment analysis revealed an overrepresentation of genes significantly correlated with *PGRMC1* that were related to mitochondrial dysfunction, ubiquitination, DNA damage, and oxidative phosphorylation pathways (Supplementary Fig. S5B).

### Prolif224 is strongly related to PAM50, and the prognostic value is similar to the current clinical standard-of-care recurrence risk scores

**Figure 4:**
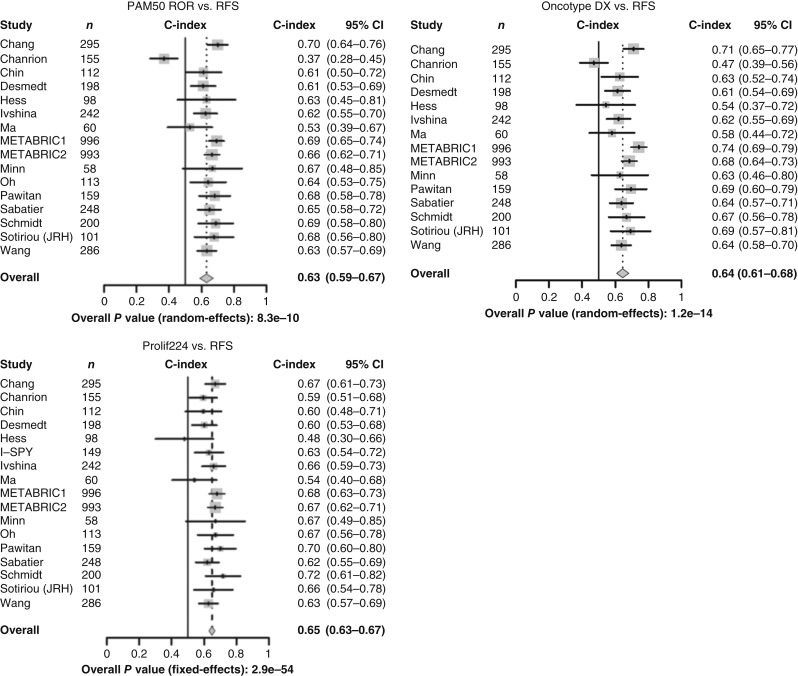
PAM50, derived Oncotype DX, and a new proliferation score (Prolif224) were all similarly related to RFS. JRH, John Radcliffe Hospital; RFS, recurrence-free survival.

We tested whether our proliferation score, Prolif224, was related to Oncotype DX and/or PAM50. We hypothesized that there would be some correlation because each recurrence score has proliferation as a strong component. Prolif224 was strongly related to PAM50 ROR (0.82, *P* = 5.7 × 10^−36^) and greatest in ER+/HER2− (*r* = 0.85; *P* = 1.5 × 10^−157^) and HER2− disease (*r* = 0.86; *P* = 0; Supplementary Table S5). The correlation with Oncotype DX was somewhat weaker at 0.7 overall (*P* = 1.4 × 10^−30^; Supplementary Table S5). We tested a derived PAM50, Oncotype DX score, and our proliferation signature as predictive biomarkers. This established the predictive value of our signature as compared with standard clinical risk scores. The concordance index (C-Index) is a commonly used metric for assessing the association between a continuous variable (e.g., signature scores) and time-to-recurrence data. It is not affected by the scale of continuous variables and deals with censored observations. It was used in the Sage Bionetworks–DREAM Breast Cancer Prognosis Challenge (79), in which the best model among 300 international teams achieved a C-Index of ∼0.75. In that context, the research version of the 70-gene MammaPrint signature was reported to have a C-Index of ∼0.6. The CIs for PAM50, Oncotype DX, and our new proliferation signature were from 0.63 to 0.66, which is moderate and reasonable, consistent with these prior reported data (Fig. 4). LDLR and PGRMC1 did not demonstrate a moderate or strong correlation with PAM50 ROR. TK1 and TMEM97 were moderately to strongly correlated. In both cases when comparing ER+, ER−, ER+/HER2−, and ER−/HER2−, the correlation was strongest in ER+/HER2− disease (TK1: *r* = 0.70; *P* = 6.4 × 10^−105^ and TMEM97: *r* = 0.46; *P* = 2.1 × 10^−137^; Supplementary Table S6).

## Discussion

We report for the first time an analysis of the individual components of the putative PGRMC1–σ2R/TMEM97–LDLR complex in human breast cancer as a function of receptor subtype, molecular subtype, and proliferation. Our studies reveal that each component is differentially expressed across breast cancer molecular subtypes, with the highest levels of expression observed within ER− disease. In addition, each protein in the complex has higher expression in high-grade tumors, and all three are positively associated with tumor cell proliferation rates, with the strongest association seen with TMEM97. Furthermore, we demonstrate that PGRMC1 and TMEM97 expression are associated with an increased risk of tumor recurrence within the first 5 years following breast cancer diagnosis, in a manner that seems to be mediated by their association with cellular proliferation. In the case of TMEM97, this is only applicable in ER+ disease. Our prognostic findings are supported by prior work noting that PGRMC1 is associated with tumor aggressiveness (14, 80, 81) and an analysis of a small patient subset (69 tumors) demonstrating that PGRMC1 overexpression is associated with breast cancer recurrence and decreased survival when untreated tumor expression is dichotomized into positive and negative PGRMC1 IHC staining (82). There is also prior work demonstrating that patients with increased PGRMC1 have decreased overall survival (HR = 1.7; *P* = 0.029; ref. 83), but the latter publication did not account for the association of PGRMC1 with proliferation, which is known to correlate with worse survival outcomes in patients with breast cancer.

The most important finding in our study is that the impact of TMEM97 on recurrence-free survival seems to be mediated by its association with proliferation. This suggests a mechanism to explain a decade of data demonstrating that σ2R/TMEM97 correlates with worse outcomes in a variety of solid tumors, including gastric (84), non–small cell lung (85, 86), squamous cell lung (87), and ovarian cancers (88). The association of TMEM97 with proliferation in breast cancer is consistent with *in vitro* cell culture studies (33, 89, 90) as well as studies in mice utilizing a highly selective, optically labeled (fluorescent) σ2R ligand probe, SW120, wherein SW120 binding was positively correlated with the cell proliferation marker Ki-67 (91). Additionally, the association of σ2R/TMEM97 with proliferation indicates that the σ2R-selective *in vivo* radioligand imaging agent ^18^F-ISO-1 may be a useful marker for breast cancer imaging that could have utility in targeted cell-cycle therapy selection and evaluating response to therapy. Supporting this, a clinical trial correlated ^18^F-ISO-1 uptake *in vivo* with Ki-67 in ER+ breast cancer (39), notably the same IHC subset in which the correlation between σ2R/TMEM97 and proliferation is the strongest and the same subset in which the association with early relapse is the highest. In particular, ^18^F-ISO-1 may provide information distinct from, and possibly complementary to another novel radiotracer, ^18^F-FLT. Unlike Ki-67 and ^18^F-ISO-1, ^18^F-FLT is trapped exclusively during the S-phase and not during G_1_, M, or G_2_. Furthermore, ^18^F-FLT has high background uptake in bone marrow, making it impossible to monitor bone metastasis, which is especially important in patients with breast cancer with receptor-positive disease. In an early human study for ^18^F-ISO-1, bone marrow uptake was noted to be low, making this a possible imaging agent for bone metastasis (34).

Table 1 shows an association between PGRMC1, TMEM97 and proliferation. This is consistent with previous literature showing high expression of these two proteins in rapidly proliferating cells. However, Table 1 also reveals only a weak correlation between LDLR and proliferation. As these proteins form a ternary complex, it is important to explain why all three proteins are not strongly correlated with proliferation. We propose the following explanation: The Nobel Prize in Physiology or Medicine in 1985 was awarded jointly to Michael S. Brown and Joseph L. Goldstein for their discoveries about the regulation of cholesterol metabolism, which includes LDLR-mediated internalization. In normal tissues and quiescent tumor cells, this mechanism explains how cells take up LDL. However, in proliferating tumor cells, the demand for cholesterol surpasses the capacity of the Brown and Goldstein mechanism.

**Figure 5:**
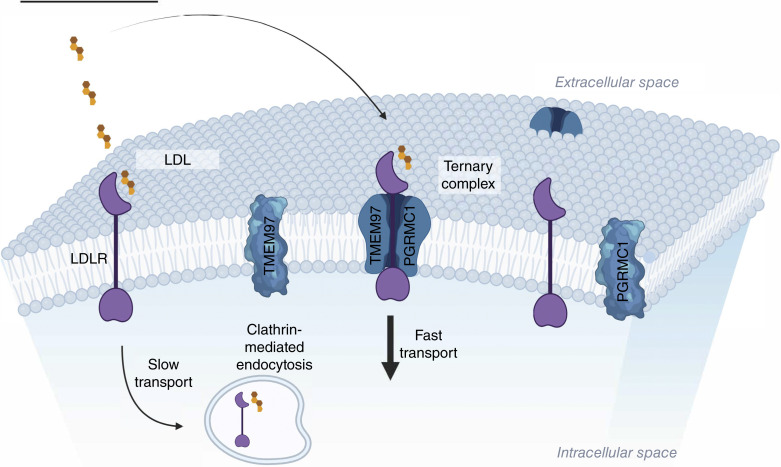
Proposed mechanism of a secondary pathway for cholesterol internalization utilized by rapidly proliferating cells demonstrating an “accelerated rate mechanism” of receptor mediated endocytosis. Created in BioRender. McDonald, E. (2024) https://BioRender.com/i39x933.

As a result, tumor cells have developed an alternative mechanism that increases the internalization rate of LDL. This is when the sigma-2–PGRMC1–LDL complex becomes crucial, as it can enhance the rate of internalization by up to tenfold (Fig. 5). *Thus*, *we propose a revision to the Brown and Goldstein mechanism to include a secondary pathway for cholesterol internalization utilized by rapidly proliferating cells*. We refer to this as the “skip-the-line” mechanism because the ternary complex offers cholesterol a modified pathway of receptor mediated endocytosis to provide the heightened demand for cholesterol to support cell proliferation. TMEM97 and PGRMC1 are upregulated whereas the LDLR is not since the balance between the Brown and Goldstein mechanism and the "skip the line" mechanism is determined by the density of TMEM97 and PGRMC1 in the cell membrane. This observation aligns with prior studies showing that the activation of LDLR-mediated cholesterol influx is linked to cancer cell growth (92). The mechanisms underlying cholesterol biosynthesis and uptake in relation to cancer progression remain largely unclear. Therefore, further mechanistic studies, both *in vivo* and *in vitro*, are needed in addition to population-based epidemiologic data to better understand the role of cholesterol in cancer development.

The second important finding is that PGRMC1 is associated with proliferation. The clinical significance of the association of PGRMC1 with proliferation includes the ongoing investigations into why one arm of the Women’s Health Initiative, including women treated with combination estrogen/progestin, had an increased risk for developing breast cancer versus the estrogen-only arm (93). PGRMC1 involvement in steroidogenesis, P4 responses in the nervous system, and cells associated with the female reproductive system are extensively established (94–96), and it has been postulated that PGRMC1 mediated the increased risk of breast cancer in the estrogen/progesterone arm via activation by synthetic progestin. There is evidence from cultured breast cancer cells and xenograft studies in mice to support this hypothesis (13, 16, 97–99). Interestingly, PGRMC1 shows a stronger correlation with proliferation in ER− cells, whereas TMEM97 is more closely associated with proliferation in ER+ cells. This raises questions about how ER status fits into the broader context of tumor proliferation and cholesterol transport. Notably, TMEM97 is generally more strongly correlated with proliferation, and variations in proliferation rates are likely more significant in ER+ tumors, as these tumors exhibit a wide range of proliferation rates that can influence both treatment response and survival outcomes. One potential mechanism by which the more aggressive ER+ subgroup may overcome barriers to proliferation could involve cholesterol transport, a mechanism that might be less critical in ER− tumors. In contrast, TNBC tumors tend to have more uniform proliferation rates.

In a prior publication (36), we demonstrated in cells that there is more PGRMC1 not complexed with TMEM97 than TMEM97 not complexed with PGRMC1. This observation suggests that PGRMC1 performs multiple functions within the cell, with its complex formation with TMEM97 and the LDLR representing just one of its roles. In contrast, TMEM97 may primarily function in conjunction with PGRMC1 and LDLR to facilitate LDL transport, which could explain why TMEM97 has a stronger correlation with proliferation. This hypothesis warrants further investigation, as the specific functions of both proteins remain poorly understood. The role of PGRMC1 in supporting increased cholesterol demand may be more tightly tied to proliferation than the other functions of PGRMC1.

The role of cholesterol trafficking in the proliferation of human breast cancer is poorly understood, with some evidence that SERM inhibit angiogenesis independent of ERs, with that mechanism being partially attributed to inhibiting cholesterol trafficking in endothelial cells (9). Our data demonstrate that differential expression of PGRMC1 in human breast cancer is a function of cell proliferation, as well as breast cancer receptor and molecular subtypes, and further reveal an association between PGRMC1 and cell-cycle markers. Although the mechanisms underlying the association of PGRMC1 with proliferation are unknown, potential effector pathways associated with PGRMC1 expression in breast cancer provide a possible explanation (Supplementary Table S4). For example, increased cyclin D1 and MYC pathway activities were each correlated with PGRMC1 expression. *Cyclin D1* is an oncogene that is frequently amplified in human breast cancer, regulates cell-cycle progression, and is associated with chemoresistance (100) and decreased overall survival in patients with ER+ breast cancers (101). Like *cyclin D1*, *c-MYC* is an oncogene that regulates cell growth and cell proliferation at the G_1_ transition (102), and its amplification is associated with aggressive tumor behavior and poor outcome in patients with breast cancer (103). PGRMC1 was also associated with fourfold lower levels of RICTOR pathway activity. RICTOR is a subunit of the mTOR complex 2 that promotes proliferation through Akt/PKB signaling (104), which in turn regulates mTORC1, a cell-cycle progression factor implicated in resistance to endocrine therapy (105, 106).

A strength of this study is that publicly available data were leveraged to analyze a large number of invasive breast cancers, with the power to detect correlations that can guide further studies at the protein level. Limitations of our study include that mRNA expression may not accurately reflect protein levels, which have greater biological significance, that proteins may undergo posttranslational modifications, such as phosphorylation, that could affect ligand binding, and that protein subcellular localization might differ in tumors compared with normal tissue.

In summary, each component of the PGRMC1, TMEM97, and LDLR complex is a breast cancer biomarker associated with cellular proliferation. This should help guide *in vitro* and *in vivo* studies exploring them in the context of additional markers of proliferation. These data also inform the clinical use of ^18^F-ISO-1 in breast cancer, in which ^18^F-ISO-1 correlated with Ki-67, providing independent clinical trial data supporting the association of a component of the trimeric complex with breast cancer proliferation (39). ^18^F-FLT and ^18^F-ISO-1 PET/CT have the potential to serve as a clinically translatable approach for predicting and monitoring response to combinatorial CDK4/6 inhibitors and endocrine therapy in patients with ER+ breast cancer, with ^18^F-FLT measuring immediate changes in the S-phase as a predominate effect of targeting CDK4/6, providing a very early prediction of tumor response, and ^18^F-ISO-1 assessing delayed changes reflecting cell-cycle arrest and transition to quiescence (35). This work exploring the role of PGRMC1–TMEM97–LDLR in breast cancer demonstrates the importance of further research evaluating how proliferation interplays with cholesterol metabolism in malignant transformation or propagation.

## Supplementary Material

Supplemental Figure S1 This shows PGRMC1 expression is increased in ER-, PR-, and triple negative human breast cancers.

Supplemental Figure S2 This shows the association of TK1 with early breast cancer relapse segregated by molecular subtype.

Supplementary Figure S3 This shows that TK1 is associated with early breast cancer relapse.

Supplemental Figure S4 This shows that the association of TK1 with breast cancer relapse is dependent on proliferation.

Supplemental Figure S5 Supplemental Figure S5

Supplemental Table S1 These are the publicly available microarray datasets combined for this analysis.

Supplemental Table S2 This shows the number of samples with available data for analysis.

Supplemental Table S3 The shows the number of samples with RNA-sequencing data available from the TCGA database.

Supplemental Table S4 This shows the upstream regulators with significant number of targets correlated with PGRMC1

Supplemental Table S5 This shows the association of our proliferation score with established scores like Oncotype Dx and PAM50.

Supplemental Table S6 This shows the trimeric complex correlation with PAM50 ROR.
