# Supplementary material for: Ternary Complex Components Responsible for Rapid LDL Internalization as Biomarkers for Breast Cancer Associated with Proliferation and Early Recurrence
Source: Cancer Res Commun. 2025 Feb 4;5(2):226–39. doi: 10.1158/2767-9764.CRC-23-0562 (PMC11791746; doi:10.1158/2767-9764.CRC-23-0562)
Supplement: Supplemental Figure S1 — This shows PGRMC1 expression is increased in ER-, PR-, and triple negative human breast cancers. [file crc-23-0562_supplemental_figure_s1_suppsf1.pdf]

<u>Supplemental Figure S1</u>: PGRMC1 expression is increased in ER-, PR-, and triple negative human breast cancers. RPKM = expression calculated as number of Reads per 1Kb of transcript per 1 Million of sequenced reads in a sample.

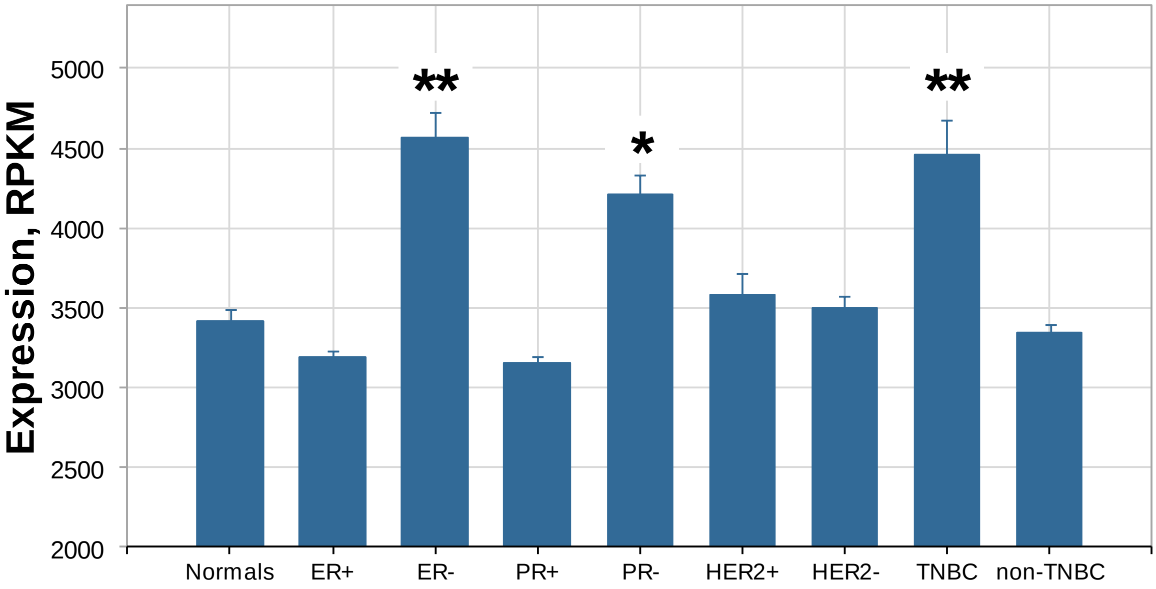

*p<0.001, **p<0.00001 for comparison of tumor versus normal tissue
