# Supplementary material for: Ternary Complex Components Responsible for Rapid LDL Internalization as Biomarkers for Breast Cancer Associated with Proliferation and Early Recurrence
Source: Cancer Res Commun. 2025 Feb 4;5(2):226–39. doi: 10.1158/2767-9764.CRC-23-0562 (PMC11791746; doi:10.1158/2767-9764.CRC-23-0562)

**Supplemental Figure S2: Association of *TK1* with early breast cancer relapse segregated by molecular subtype.** Effect size estimates were aggregated across data sets by meta-analysis to determine risk of relapse within 5 years from all cancers.

**a) Association of *TK1* and early breast cancer relapse in all molecular subtypes.**

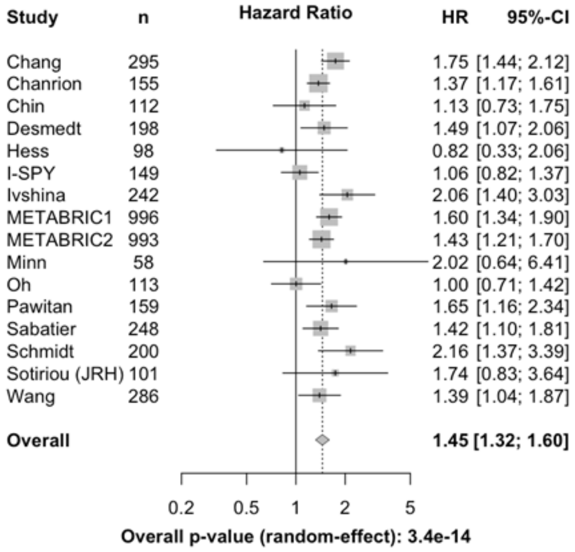


**b) Association of *TK1* and early breast cancer relapse in the luminal A subtype.**

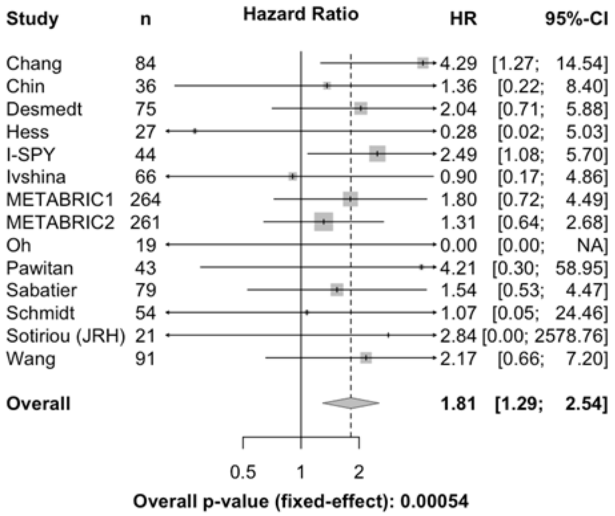


**c) Association of *TK1* and early breast cancer relapse in the basal subtype.**

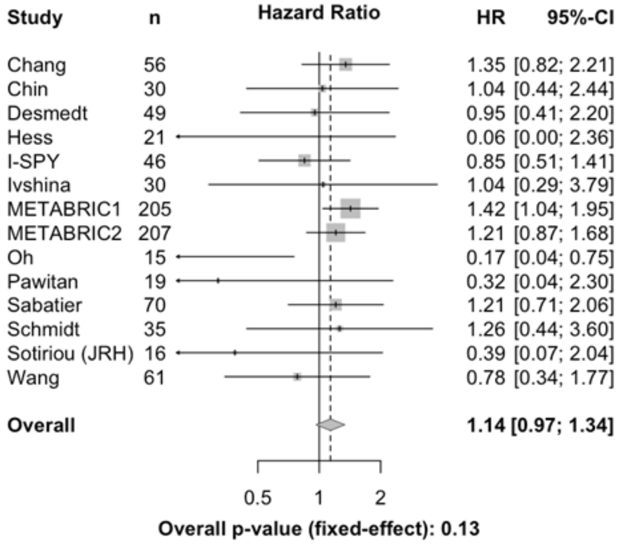

Supplement: Supplemental Figure S2 — This shows the association of TK1 with early breast cancer relapse segregated by molecular subtype. [file crc-23-0562_supplemental_figure_s2_suppsf2.pdf]
