# Supplementary material for: Ternary Complex Components Responsible for Rapid LDL Internalization as Biomarkers for Breast Cancer Associated with Proliferation and Early Recurrence
Source: Cancer Res Commun. 2025 Feb 4;5(2):226–39. doi: 10.1158/2767-9764.CRC-23-0562 (PMC11791746; doi:10.1158/2767-9764.CRC-23-0562)

<u>Supplemental Figure S3</u>: Association of *TK1* with early breast cancer relapse segregated by IHC status. Effect size estimates were aggregated across data sets by meta-analysis to determine risk of relapse within 5 years from all cancers.

a) Association of *TK1* and early breast cancer relapse in ER+/HER2- tumors.

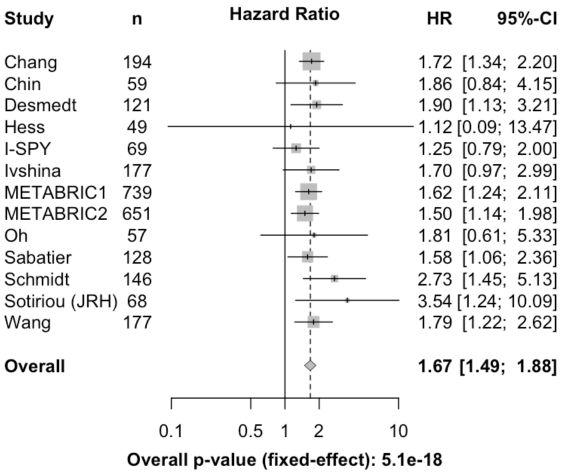

b) Association of *TK1* and early breast cancer relapse in ER+ tumors.

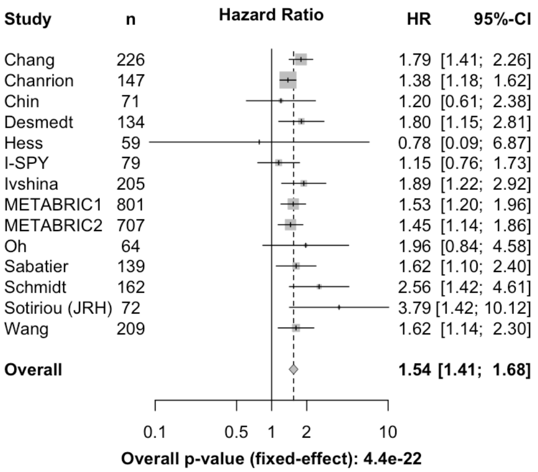

c) Association of *TK1* and early breast cancer relapse in HER2- tumors.

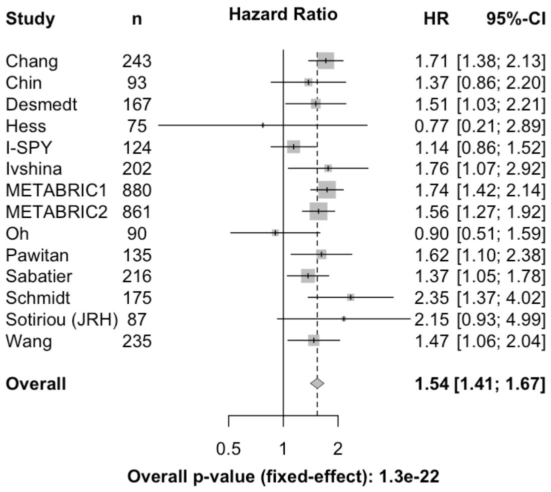

Supplement: Supplementary Figure S3 — This shows that TK1 is associated with early breast cancer relapse. [file crc-23-0562_supplementary_figure_s3_suppsf3.pdf]
