# Supplementary material for: Ternary Complex Components Responsible for Rapid LDL Internalization as Biomarkers for Breast Cancer Associated with Proliferation and Early Recurrence
Source: Cancer Res Commun. 2025 Feb 4;5(2):226–39. doi: 10.1158/2767-9764.CRC-23-0562 (PMC11791746; doi:10.1158/2767-9764.CRC-23-0562)

<u>Supplemental Figure S4</u>: Association of *TK1* with early breast cancer relapse adjusted for estimated proliferation. Effect size estimates were aggregated across data sets by meta-analysis to determine risk of relapse within 5 years from all cancers.

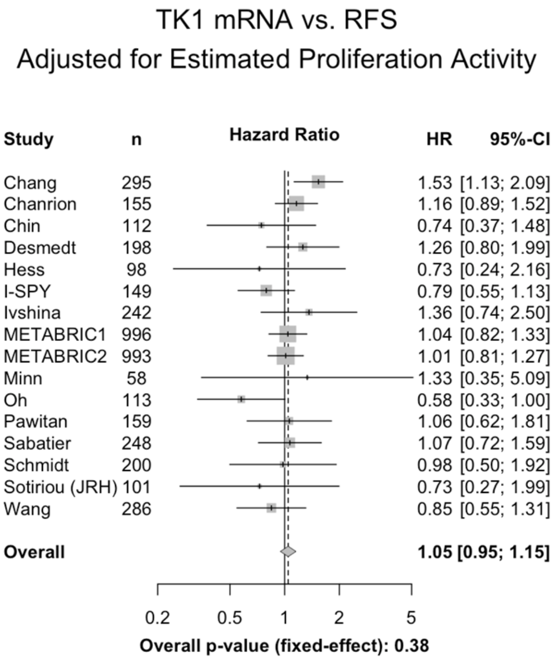

Supplement: Supplemental Figure S4 — This shows that the association of TK1 with breast cancer relapse is dependent on proliferation. [file crc-23-0562_supplemental_figure_s4_suppsf4.pdf]
