# Supplementary material for: Ternary Complex Components Responsible for Rapid LDL Internalization as Biomarkers for Breast Cancer Associated with Proliferation and Early Recurrence
Source: Cancer Res Commun. 2025 Feb 4;5(2):226–39. doi: 10.1158/2767-9764.CRC-23-0562 (PMC11791746; doi:10.1158/2767-9764.CRC-23-0562)
Supplement: Supplemental Figure S5 [file crc-23-0562_supplemental_figure_s5_suppsf5.pdf]

Supplemental Figure S5A: Gene expression and pathways associated with *PGRMC1*. A) Representative heat map for gene expression correlated with *PGRMC1*. Saturated red = highest expression (2 fold or more over average), saturated blue = lowest expression (2 fold or more below average) with shades of blue and red in between. Corr = Pearson correlation coefficient with expression of *PGRMC1*.

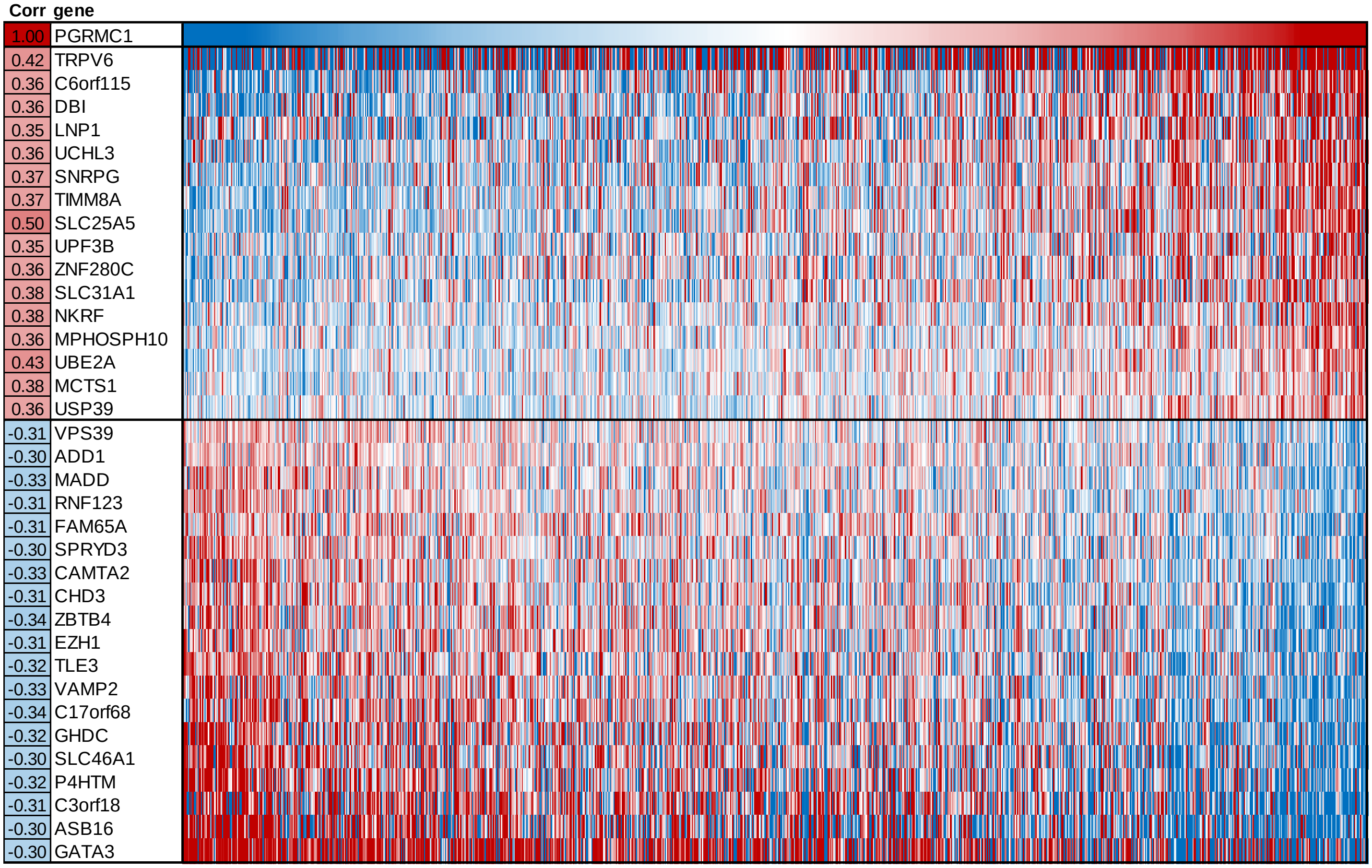

Supplemental Figure S5B: Pathways enrichment analysis for gene expression significantly correlated with *PGRMC1*.

Canonical Pathway	*P*	FDR	N	pos	neg	Molecules (correlation *r* with PGRMC1)
**Mitochondrial Dysfunction**	7 x 10^{-5}	2%	13	12	1	COX7B(0.29), NDUFA9(0.27), CYCS(0.27), UQCRH(0.31), GLRX2(0.29), NDUFA1(0.29), BCL2(-0.26), PDHA1(0.26), ATP5C1(0.29), ATP5J2(0.25), ATP5F1(0.26), ATP5G3(0.27), AIFM1(0.29)
**Protein Ubiquitination Pathway**	0.0001	2%	16	15	1	UCHL3(0.36), CDC20(0.25), HSPA14(0.28), UBE2A(0.43), DNAJC2(0.33), USP1(0.27), USP39(0.36), UBE2D1(0.27), PAN2(-0.29), PSMD10(0.30), PSMB2(0.29), PSMA5(0.25), PSMD14(0.26), PSMA3(0.25), UBE2E3(0.35), PSMC2(0.30)
**Oxidative Phosphorylation**	0.0005	5%	9	9	0	ATP5C1(0.29), COX7B(0.29), NDUFA9(0.27), CYCS(0.27), UQCRH(0.31), ATP5J2(0.25), NDUFA1(0.29), ATP5F1(0.26), ATP5G3(0.27)
**Nur77 Signaling in T Lymphocytes**	0.0012	9%	6	4	2	CALM1(0.27), HDAC2(0.32), CYCS(0.27), PPP3R1(0.33), MEF2D(-0.27), BCL2(-0.26)
**Arsenate Detoxification I (Glutaredoxin)**	0.0026	16%	2	2	0	GLRX2(0.29), PNP(0.29)
**Mismatch Repair in Eukaryotes**	0.0043	21%	3	3	0	MSH2(0.25), RFC2(0.31), EXO1(0.28)
**DNA Methylation and Transcriptional Repression Signaling**	0.0081	33%	3	2	1	CHD3(-0.31), HDAC2(0.32), SAP30(0.26)
**Inositol Pyrophosphates Biosynthesis**	0.0087	33%	2	0	2	IP6K1(-0.29), PPIP5K1(-0.26)
**Pyrimidine Deoxyribonucleotides De Novo Biosynthesis I**	0.010	34%	3	3	0	RRM2(0.26), AK4(0.28), CMPK1(0.30)
**Role of BRCA1 in DNA Damage Response**	0.011	34%	5	5	0	MSH2(0.25), RFC2(0.31), E2F3(0.28), FANCL(0.25), CHEK2(0.26)

Canonical Pathway: canonical pathway enriched; P: nominal p-value of the enrichment; FDR: false discovery rate - p-value corrected for multiple testing according to Benjamini-Hochberg procedure; Total: total known number of genes in the pathway; N: number of genes from the analyzed list in the pathw ay; pos: number of molecules positively correlated with PGRMC1; neg: number of molecules negatively correlated with PGRMC1; Molecules: genes or complexes from the gene list involved in the pathway with correlation with PGRMC1 in parenthesis.
