# Supplementary material for: Ternary Complex Components Responsible for Rapid LDL Internalization as Biomarkers for Breast Cancer Associated with Proliferation and Early Recurrence
Source: Cancer Res Commun. 2025 Feb 4;5(2):226–39. doi: 10.1158/2767-9764.CRC-23-0562 (PMC11791746; doi:10.1158/2767-9764.CRC-23-0562)
Supplement: Supplemental Table S1 — These are the publicly available microarray datasets combined for this analysis. [file crc-23-0562_supplemental_table_s1_suppst1.pdf]

<u>Supplemental Table S1</u>: Integrated Dataset. Publicly available microarray datasets combined for this analysis

Study	Sample Size	Hormonal Status	No Systemic Treatment	Neoadjuvant Treatment	Event Types
Chang	295	Mixed			Relapse
Chanrion	155	Positive			Relapse
Chin	112	Mixed			Relapse
Desmedt	198	Mixed	x		Relapse
Hess	98	Mixed		x	Relapse
I-SPY	149	Mixed		x	Relapse
Ivshina	242	Mixed			Relapse
Ma	60	Positive			Relapse
METABRIC1	996	Mixed			Disease-Specific Death
METABRIC2	993	Mixed			Disease-Specific Death
Minn	58	Negative	x		Metastasis
Oh	113	Mixed			Relapse
Pawitan	159	Mixed			Relapse
Sabatier	248	Mixed			Relapse
Schmidt	200	Mixed	x		Metastasis
Sotiriou (JRH)	101	Mixed			Relapse
Wang	286	Mixed	x		Metastasis
