# Supplementary material for: Ternary Complex Components Responsible for Rapid LDL Internalization as Biomarkers for Breast Cancer Associated with Proliferation and Early Recurrence
Source: Cancer Res Commun. 2025 Feb 4;5(2):226–39. doi: 10.1158/2767-9764.CRC-23-0562 (PMC11791746; doi:10.1158/2767-9764.CRC-23-0562)
Supplement: Supplemental Table S2 — This shows the number of samples with available data for analysis. [file crc-23-0562_supplemental_table_s2_suppst2.pdf]

<u>Supplemental Table S2</u>: Number of samples with available data

Strata	All Samples	No Systemic Treatment
All	4463	1649
ER+	3075	1165
ER-	1100	446
HER2+	606	220
HER2-	3583	1371
ER+/HER2+	283	113
ER+/HER2-	2635	1051
ER-/HER2+	277	98
ER-/HER2-	764	290

Strata	All Samples	No Systemic Treatment
Node+	1627	55
Node-	2546	1583
Basal	860	319
ERBB2	645	235
Luminal B	921	296
Luminal A	1164	505
Normal-like	543	236
