# Supplementary material for: Ternary Complex Components Responsible for Rapid LDL Internalization as Biomarkers for Breast Cancer Associated with Proliferation and Early Recurrence
Source: Cancer Res Commun. 2025 Feb 4;5(2):226–39. doi: 10.1158/2767-9764.CRC-23-0562 (PMC11791746; doi:10.1158/2767-9764.CRC-23-0562)
Supplement: Supplemental Table S3 — The shows the number of samples with RNA-sequencing data available from the TCGA database. [file crc-23-0562_supplemental_table_s3_suppst3.pdf]

**Supplemental Table S3: Number of samples with RNA-sequencing data available from the TCGA database.**

| Tissue | subtype | N samples |
|---|---|---|
| Tumor | ER+ | 738 |
| | ER- | 215 |
| | PR+ | 643 |
| | PR- | 307 |
| | HER2+ | 149 |
| | HER2- | 508 |
| | TNBC | 102 |
| | not-TNBC | 550 |
| | all | 1019 |
| Normal | all | 108 |
