# Supplementary material for: Ternary Complex Components Responsible for Rapid LDL Internalization as Biomarkers for Breast Cancer Associated with Proliferation and Early Recurrence
Source: Cancer Res Commun. 2025 Feb 4;5(2):226–39. doi: 10.1158/2767-9764.CRC-23-0562 (PMC11791746; doi:10.1158/2767-9764.CRC-23-0562)
Supplement: Supplemental Table S4 — This shows the upstream regulators with significant number of targets correlated with PGRMC1 [file crc-23-0562_supplemental_table_s4_suppst4.pdf]

**Supplemental Table S4: Upstream regulators with significant number of targets correlated with *PGRMC1*.** P = p value of the regulator targets enrichment among the list of 416 genes correlated with *PGRMC1*. Z = z-score of the predicted regulator activity based on targets direction of correlation with *PGRMC1*. Red = most likely activated with increase of *PGRMC1*, Blue = most likely inhibited.

| Type | Regulator | *p* | Z |
|---|---|---|---|
| transcription regulator | CCND1 | 8E-05 | 2.45 |
| transcription regulator | MYC | 1E-06 | 1.95 |
| transcription regulator | TP53 | 3E-06 | 0.74 |
| chemical drug | CD437 | 3E-06 | -4 |
| complex | CD3 | 1E-05 | -2 |
| other | RICTOR | 3E-05 | -4 |
