# Supplementary material for: Ternary Complex Components Responsible for Rapid LDL Internalization as Biomarkers for Breast Cancer Associated with Proliferation and Early Recurrence
Source: Cancer Res Commun. 2025 Feb 4;5(2):226–39. doi: 10.1158/2767-9764.CRC-23-0562 (PMC11791746; doi:10.1158/2767-9764.CRC-23-0562)
Supplement: Supplemental Table S5 — This shows the association of our proliferation score with established scores like Oncotype Dx and PAM50. [file crc-23-0562_supplemental_table_s5_suppst5.pdf]

Supplemental Table 5

Proliferation vs Pam50_ROR

Strata	Correlation Coefficient	p-value
All	0.819	5.7e-36
ER+	0.821	2.1e-24
ER-	0.762	1.8e-102
HER2+	0.735	3e-106
HER2-	0.864	0
ER+/HER2+	0.781	6.6e-61
ER+/HER2-	0.847	1.5e-157
ER-/HER2+	0.746	5.7e-48
ER-/HER2-	0.811	4e-190
Node+	0.814	9.9e-17
Node-	0.819	4.8e-21
G1&G2	0.78	2.3e-28
G3	0.764	3.4e-29
Basal	0.685	1.9e-120
ERBB2+	0.683	8.5e-91
Luminal A	0.526	2.1e-82
Luminal B	0.698	2e-140
Normal-like	0.597	1.8e-53

Proliferation vs OncotypeDX

Strata	Correlation Coefficient	p-value
All	0.697	1.4e-30
ER+	0.618	2.1e-17
ER-	0.67	5.4e-57
HER2+	0.45	1.5e-28
HER2-	0.749	6.8e-79
ER+/HER2+	0.533	2e-20
ER+/HER2-	0.655	3.1e-34
ER-/HER2+	0.408	1.9e-10
ER-/HER2-	0.777	1.1e-153
Node+	0.658	1.2e-10
Node-	0.696	1.3e-40
G1&G2	0.541	9e-14
G3	0.638	3.5e-42
Basal	0.587	9.9e-76
ERBB2+	0.477	8.7e-36
Luminal A	0.062	0.34
Luminal B	0.623	4.6e-40
Normal-like	0.234	0.023
