# Supplementary material for: Ternary Complex Components Responsible for Rapid LDL Internalization as Biomarkers for Breast Cancer Associated with Proliferation and Early Recurrence
Source: Cancer Res Commun. 2025 Feb 4;5(2):226–39. doi: 10.1158/2767-9764.CRC-23-0562 (PMC11791746; doi:10.1158/2767-9764.CRC-23-0562)
Supplement: Supplemental Table S6 — This shows the trimeric complex correlation with PAM50 ROR. [file crc-23-0562_supplemental_table_s6_suppst6.pdf]

Supplemental Table 6

LDLR vs Pam50_ROR

Strata	Correlation Coefficient	p-value
All	0.127	6e-17
ER+	0.115	3.2e-10
ER-	-0.009	0.77
HER2+	0.066	0.12
HER2-	0.091	1e-07
ER+/HER2+	0.135	0.033
ER+/HER2-	0.077	0.00011
ER-/HER2+	-0.064	0.34
ER-/HER2-	-0.013	0.73
Node+	0.107	3.2e-05
Node-	0.134	2.4e-11
G1&G2	0.13	1.3e-09
G3	0.056	0.018
Basal	-0.057	0.11
ERBB2+	0.087	0.035
Luminal A	0.008	0.79
Luminal B	0.009	0.79
Normal-like	0.06	0.18

PGRMC1 vs Pam50_ROR

Strata	Correlation Coefficient	p-value
All	0.101	0.028
ER+	-0.034	0.49
ER-	0.065	0.039
HER2+	-0.006	0.88
HER2-	0.128	0.00054
ER+/HER2+	-0.062	0.33
ER+/HER2-	-0.032	0.43
ER-/HER2+	-0.033	0.62
ER-/HER2-	0.109	0.0043
Node+	0.092	0.18
Node-	0.081	0.12
G1&G2	-0.005	0.92
G3	0.106	0.016
Basal	0.136	0.00014
ERBB2+	0.124	0.0025
Luminal A	-0.167	3.1e-08
Luminal B	-0.019	0.58
Normal-like	-0.107	0.016

TK1 vs Pam50_ROR

Strata	Correlation Coefficient	p-value
All	0.629	1.3e-16
ER+	0.622	1.9e-12
ER-	0.567	6.9e-36
HER2+	0.481	3.3e-34
HER2-	0.706	4.8e-157
ER+/HER2+	0.507	5.2e-15
ER+/HER2-	0.696	6.4e-105
ER-/HER2+	0.395	2.7e-10
ER-/HER2-	0.606	1.2e-29
Node+	0.566	6e-06
Node-	0.652	3.8e-19
G1&G2	0.613	3.5e-13
G3	0.533	1.1e-19
Basal	0.44	1.1e-15
ERBB2+	0.375	1.2e-21
Luminal A	0.428	3.5e-51
Luminal B	0.428	8.6e-41
Normal-like	0.435	1.8e-25

TMEM97 vs Pam50_ROR

Strata	Correlation Coefficient	p-value
All	0.351	1.7e-08
ER+	0.417	3.2e-08
ER-	0.295	6.5e-21
HER2+	0.227	1.2e-07
HER2-	0.404	3e-135
ER+/HER2+	0.265	2.3e-05
ER+/HER2-	0.463	2.1e-137
ER-/HER2+	0.268	5.5e-05
ER-/HER2-	0.336	5.9e-19
Node+	0.309	9e-04
Node-	0.357	1.7e-10
G1&G2	0.342	4.7e-07
G3	0.245	1.2e-06
Basal	0.159	0.015
ERBB2+	0.211	2.6e-07
Luminal A	0.189	4e-10
Luminal B	0.355	4.2e-27
Normal-like	0.215	1.1e-06
